# HMGA1B/2 transcriptionally activated-POU1F1 facilitates gastric carcinoma metastasis via CXCL12/CXCR4 axis-mediated macrophage polarization

**DOI:** 10.1038/s41419-021-03703-x

**Published:** 2021-04-29

**Authors:** Cheng Tang, Xiong Lei, Lingqiang Xiong, Zhigao Hu, Bo Tang

**Affiliations:** General surgery department, The First Affiliated Hospital of Nanchang University, 330006 Nanchang, Jiangxi Province P.R. China

**Keywords:** Gastric cancer, Gastric cancer

## Abstract

Tumor-associated macrophages (TAMs) in the tumor microenvironment contribute to poor prognosis in gastric cancer (GC). However, the underlying mechanism by which TAMs promote GC progression and metastasis remains elusive. Expression of POU1F1 was detected in 60 matched GC-normal tissue pairs using qRT-PCR and immunohistochemistry (IHC) analysis. The correlation between POU1F1 and the clinical-pathological factors of GC patients were further assessed. Cell proliferation was monitored by CCK-8, colony formation, and 5-Ethynyl-2’-deoxyuridine (EdU) incorporation assays. Cell migration and invasion were assessed by transwell assays. The impact on angiogenesis was evaluated by tube formation assay. Xenograft model was generated to investigate the role of POU1F1 on tumor growth and lung metastasis in vivo. GST pull-down and Co-immunoprecipitation (Co-IP) were used to study the interaction between HMGA1B/2 and POU1F1. Chromatin immunoprecipitation (ChIP) and dual luciferase reporter assays were performed to investigate the transcriptional regulation of POU1F1. Flow cytometry was performed to detect the surface expression of macrophage markers. Upregulated POU1F1 observed both in GC tissues and cell lines was positively correlated with poor prognosis. Knockdown of POU1F1 inhibited cell proliferation, migration, invasion, and angiogenesis in vitro, and suppressed tumor growth in vivo. HMGA1B/2 transcriptionally activated-POU1F1. POU1F1 promoted GC progression via regulating macrophage proliferation, migration, polarization, and angiogenesis in a CXCL12/CXCR4-dependent manner. POU1F1 also promoted GC metastasis in lung by modulating macrophage polarization through CXCL12/CXCR4 axis in vivo. HMGA1B/2-upregulated POU1F1 promoted GC metastasis via regulating macrophage polarization in a CXCL12/CXCR4-dependent manner.

## Introduction

Gastric cancer (GC) is one of the most common diagnosed cancer worldwide^1^. Unfortunately, the 5-year survival rate for patients with GC remains unfavorable due to local relapse or distant metastasis^2^. An increasing body of literature illustrates that tumor microenvironment contributes to tumor growth and metastasis^3–5^. Tumor microenvironment consists of different cell populations in which cancer cells communicate with each other, as well as stromal cells, extracellular matrix (ECM), and various infiltrating immune cells. In recent years, macrophages gain more attentions due to its critical role in the tumor microenvironment.

Circulating monocytic precursor cells are recruited into tumors and differentiated into mature macrophages. In response to microenvironment signals, macrophages can be polarized into two functional phenotypes: classically activated M1 and alternatively activated M2 macrophages^3,6^. The M1 macrophage promotes inflammatory response against bacterial or viral infections, and exerts antitumor function. By contrast, the M2 macrophage exhibits anti-inflammatory and protumorigenic function. The conversion to M2 macrophage is stimulated by cytokines or chemokines, such as IL-4, IL-10, IL-13, and CXCL12/CXCR4 axis^6–8^. Compelling evidence has revealed that tumor-associated macrophages (TAMs) closely resemble the M2 macrophages. TAMs has emerged as an important component in the tumor microenvironment, promoting tumor progression, angiogenesis, and metastasis^6,9,10^. GC patients with TAM high-infiltration had unfavorable clinical outcomes compared to those with TAM low-infiltration^11^, indicating that TAM is significantly associated with poor prognosis in GC. Targeting TAM can be a promising therapy for GC patients. It is urgent to dissect the underlying mechanism by which TAMs exert the oncogenic role in GC.

POU class 1 homeobox 1 (POU1F1), also known as Pit-1, was originally identified in the pituitary gland and involved in pituitary gene transcription^12^. In breast cancer, xenograft study has illustrated that POU1F1 overexpression promotes tumor growth and metastasis in lung^13^. Recent mechanistic study has revealed that high POU1F1 expression in breast cancer patients is positively correlated with metastasis in liver and lung, and POU1F1/CXCL12/CXCR4 axis is involved in macrophage recruitment and polarization, as well as metastatic process^14,15^. However, the expression and function of POU1F1 in GC remains unclear.

High mobility group A (HMGA) non-histone chromatinic proteins modulate gene transcription by altering chromatin structure. HMGA family comprises HMGA1A, HMGA1B, and HMGA2 encoded by two different genes, namely *HMGA1* and *HMGA2*, respectively^16,17^. HMGA1A and HMGA1B were generated by *HMGA1* via alternative splicing. HMGA1 maintains GC cell proliferation via Wnt/β-catenin pathway^18^. HMGA2 is abundantly overexpressed in GC, and correlates with serosal invasion and poor prognosis^19^. Following mechanism studies also illustrated that HMGA2 aggravates GC metastasis via promoting epithelial-mesenchymal transition (EMT) via Wnt/β-catenin pathway^20–22^. In addition, HMGA2 was also found to promote vasculogenic mimicry via Twist-VE-cadherin signaling in GC^23^. Interestingly, HMGA1B and HMGA2 have been found to upregulate POU1F1 in pituitary cancer^24^. We thus hypothesized that HMGA-regulated POU1F1 might be involved in macrophage polarization in GC.

In this study, we demonstrated for the first time that POU1F1 was highly expressed in GC tissues and cells, which was positively correlated with poor prognosis in GC. Knockdown of POU1F1 inhibited cell proliferation, migration, invasion, and angiogenesis in vitro, and suppressed the tumor growth in vivo. Mechanistic study revealed that HMGA1B/2 transcriptionally activated-POU1F1. POU1F1 promoted GC progression via regulating macrophage proliferation, migration, polarization, and angiogenesis in a CXCL12/CXCR4-dependent manner. In vivo experiments also showed that POU1F1 promoted GC metastasis in lung by modulating macrophage polarization through CXCL12/CXCR4 axis. These findings provided novel insights into TAM-targeted therapy for GC.

## Material and methods

### Collection of clinical samples

A cohort of 60 GC tissues and paired adjacent normal tissues were collected from patients with GC postoperatively at The First Affiliated Hospital of Nanchang University from 2015 to 2019. None of the participant received preoperative treatment. This study was approved by The First Affiliated Hospital of Nanchang University and performed in accordance with the provisions of the Declaration of Helsinki and Good Clinical Practice guidelines. Written informed consents were obtained from all patients.

### Histopathological analysis

Formalin-fixed paraffin embedded (FFPE) GC tissues and paired adjacent normal tissues were sectioned, followed by staining with hematoxylin and eosin (H&E) for histopathological analysis. Immunohistochemistry (IHC) analysis was performed as described^15^. In brief, sections were deparaffinized, rehydrated, followed by antigen retrieval. After blocking with 10% normal goat serum, slides were incubated with primary antibody at 4 °C overnight. Following primary antibodies were used in IHC analysis: anti-POU1F1 (1:100; Abcam, Cambridge, MA, USA); anti-Ki67 (1:500; Abcam, Cambridge, MA, USA); CD163 (1:200; Novocastra, Laboratories, Newcastle upon Tyne, UK); CD31 (1:100; Invitrogen, Thermo Fisher Scientific, Waltham, MA, USA). The immunoreactivity of target was visualized using labeled streptavidin biotin method. The staining intensity was quantitatively analyzed using ImageJ software.

### Cell culture and transfection

Human gastric epithelial cell line GES-1 cells and gastric cancer cell lines SGC7901, BGC823, MGC803, MKN45, MKN28, AGS cells, and HUVECs were purchased from Cell Bank of Type Culture Collection, Chinese Academy of Science (Shanghai, China). Cells were maintained in DMEM or RMPI 1640 supplemented with 10% fetal bovine serum (FBS; Gibco, Thermo Fisher Scientific), 100 U/ml penicillin, and 100 μg/ml streptomycin. Human monocytic THP-1 cells were grown in RMPI 1640 containing 10% FBS, 10 mM Hepes, 1 mM pyruvate, 2.5 g/L D-glucose, and 50 pM β-mecaptoethanol (Gibco). All cell lines were cultured at 37 °C and 5% CO_2_ in humidified air.

Co-culture of GC cells and macrophages was performed using 0.4 μm tissue culture inserts (Nunc, Thermo Fisher Scientific) as previously described^15^. For CCK-8 assay, macrophages were seeded in the lower chamber, and GC cells were maintained in the upper chamber. By contrast, macrophages were grown in the upper chamber for transwell migration assay. Co-culture was performed for 24 h. For functional experiments, conditioned medium (CM) were then collected and used immediately. To remove CXCL12, CM was immunoprecipitated with protein A/G-conjugated anti-CXCL12 antibody (Santa Cruz Biotechnology, Santa Cruz, CA, USA) or normal IgG (Invitrogen) for 4 h. The flow-through was used for the subsequent experiments.

The siRNAs for POU1F1, HMGA1, HMGA2, and scramble siRNA (si-NC) were purchased from Ambion-Thermo Fisher Scientific. Expression vector containing the V5-tagged full-length cDNA for POU1F1 subcloned in the pcDNA3.1/GS vector was purchased from Invitrogen. siRNAs or overexpression constructs were transfected into cells using Lipofectamine 2000 transfection reagent (Invitrogen) according to the manufacturer’s instructions.

### RNA isolation and quantitative reverse transcriptase PCR (qRT-PCR)

Total RNA was isolated from GC tissues and cell lines using TRIzol reagent (Invitrogen) according to the manufacturer’s instructions. RNA was reverse-transcribed using QuantiTect Reverse Transcription Kit (Qiagen, Chatsworth, CA, USA). qRT-PCR was performed with SYBR Green PCR Master Mix (Applied Biosystems, Thermo Fisher Scientific). Each reaction was performed in triplicate. GAPDH was used as an internal control. The primers sequences in this study were as follows: POU1F1 forward: 5’-GTGGGAGCAAATGAAAGGAA-3’, reverse: 5’-TCACCCGTTTTTCTCTCTGC-3’; CD163 forward: 5’-TTCACTGCACTGGGACTGAG-3’, reverse: 5’- CACTCTCTATGCAGGCCACA-3’; CD31 forward: 5’- GAGAGGACATTGTGCTGCAA-3’, reverse: 5’-ATGGGGCAAGAATGACTCTG-3’; CXCL12 forward: 5’-TGAGCTACAGATGCCCATGC-3’, reverse: 5’- CCACTTTAGCTTCGGGTCAA-3’; GAPDH forward: 5’-CCAGGTGGTCTCCTCTGA-3’, reverse: 5’-GCTGTAGCCAAATCGTTGT-3’. The relative expression level of the target gene was determined using 2^-ΔΔCT^ method.

### Protein extraction and western blot

Protein lysates were extracted using RIPA lysis buffer (Pierce, Thermo Fisher Scientific). Protein estimation was conducted using a BCA protein assay kit (Pierce). Protein lysates were denatured and subjected to SDS-PAGE electrophoresis. Proteins were then transferred onto a nitrocellulose membrane and blocked with 5% non-fat milk, followed by the incubation with primary antibody at 4 °C overnight. Membranes were then rinsed and incubated with a corresponding secondary antibody (1:5000, Invitrogen) at room temperature for 1 h. Signal was visualized using Pierce ECL plus Western blotting substrate (Pierce). The following primary antibodies were used in this study: anti-POU1F1 (1:3000; Santa Cruz Biotechnology, Santa Cruz, CA, USA); anti-HA tag (1:2000; Invitrogen); anti-V5 tag (1:2000; Invitrogen); anti-CD163 (1:5000; Abcam); anti-CD206 (1:5000; Sigma–Aldrich, St Louis, MO, USA), anti-CD11b (Novus Biologicals, Centennial, CO, USA); anti-VEGFA (1:5000; Santa Cruz); anti-p-CXCR4 (1:1000; Abcam, Cambridge, UK); anti-CXCR4 (1:1000; Abcam); anti-p-Akt (1:1000; Cell signaling technology, Beverly, MA, USA); anti-Akt (1:1000; Cell signaling technology); anti-p-VEGFR2 (1:1000; Cell signaling technology); anti-VEGFR2 (1:1000; Cell signaling technology); anti-GAPDH (1:3000; Santa Cruz).

### Cell counting kit-8 (CCK-8) assay

Cell proliferation was assessed using CCK-8 kit (Beyotime, Haimen, China) according to manufacturer’s instructions. In brief, cells (1 × 10^4^ cells/well) were grown in 96-well plates. Cell proliferation was monitored every 24 h post-transfection. At designated timepoints, cells were incubated with CCK-8 solution for 1 h at 37 °C. Absorbance was measured at 490 nm using a PerkinElmer microplate reader (PerkinElmer, Waltham, MA, USA). Each reaction was performed in triplicate.

### Colony formation assay

A certain number of transfected cells were cultured in six-well plates and maintained in proper media containing 10% FBS for 2 weeks. The medium was replaced every 4 days. After 2 weeks, colonies were fixed with methanol and stained with 0.1% crystal violet (Sigma–Aldrich). Colonies containing at least 50 viable cells were analyzed.

### 5-Ethynyl-2’-deoxyuridine (EdU) incorporation assay

Cells were cultured in 24-well plates. EdU incorporation assay was conducted using the Click-iT EdU Alexa Fluor 555 Imaging Kit (Molecular Probes, Thermo Fisher Scientific) according to the manufacturer’s instructions. In brief, cells were incubated with 10 μM EdU and stained with the Click-iT EdU Alexa Fluor 555 Imaging Kit. Cell nucleus was visualized by DAPI. Fluorescence signals were acquired by Olympus confocal laser scanning microscope (Olympus Corp., Tokyo, Japan).

### Transwell migration and invasion assay

For the transwell assays, BGC823 and SGC7901 cells (5 × 10^4^ for migration or 1 × 10^5^ for invasion) were plated in 24-well transwell upper chambers (Corning Costar, NY, USA) and cultured in serum-free medium. For invasion assay, the upper chambers were precoated with Matrigel (BD Biosciences, San Jose, CA, USA). DMEM containing 10% FBS were filled in the lower chambers. After incubation with 24 h, BGC823 and SGC7901 cells remaining on the upper membrane were removed with cotton swabs. BGC823 and SGC7901 cells, which migrated or invaded through the membrane were fixed with methanol and stained with 0.1% crystal violet (Sigma–Aldrich). The migrated or invaded cells were then counted using an inverted microscope (Olympus Corp.).

### Tube formation assay

Tube formation assay was performed as previously described^14^. Briefly, HUVECs were harvested and plated onto Matrigel (BD Biosciences)-coated 24-well plates (cell density) for 24 h. Tubes were photographed using an inverted microscope (Olympus Corp.).

### ELISA assay

The levels of CXCL12 and VEGFA in cell culture medium were assessed using CXCL12 and VEGFA ELISA kits (R&D systems, Minneapolis, MN, USA) according to the manufacturer’s instructions.

### Xenograft study

BALB/c male nude mice (*n* = 7; 3–4 weeks old) were purchased from the Animal Center of the Chinese Academy of Science (Shanghai, China). All animal experiments were undertaken in accordance with the National Institute of Health guidelines for the care and use of laboratory animals, with the approval of the Ethics Committee of The First Affiliated of Nanchang University. To evaluate the tumor growth in vivo, SGC7901 cells (1 × 10^7^) stably transfected with si-NC or si-POU1F1#3 were subcutaneously injected in the upper right flank of each mouse. Tumor size was measured every 2 days using digital calipers. Tumor size was calculated using the following formula: Tumor size = lw^2^/2 (where l is the length and w is the width of tumor). After 16 days following subcutaneous inoculation, the mice were scarified, and tumors were dissected, fixed with formalin, and embedded in paraffin. To study distant metastasis, SGC7901 cells stably transfected with si-NC, si-POU1F1#3, POU1F1, and empty vector were harvested from cell culture plates, washed with PBS and resuspended at 1 × 10^7^ cells/mL. Suspended SGC7901-vector or SGC7901-POU1F1 cells (0.1 mL) combined with equal macrophages with or without MSX-122 application (10 mg/kg, i.p.) were injected into the tail veins of mice. Then, the mice were killed 7 weeks after injection. The tissues of lungs were removed and photographed, and the visible tumors on the lung surface were counted. Collected lung tissues were subjected to H&E staining and IHC analysis.

### GST pull-down assay

V5-tagged POU1F1, GST-tagged HMGA1B, and GST-tagged HMGA2 recombinant proteins were prepared as previous described^24^. GST pull-down assay was performed using Pierce GST Protein Interaction Pull-Down Kit (Pierce) according to the manufacturer’s instructions. Briefly, GST, GST-HMGA1B, or GST-HMGA2 recombinant protein was conjugated to glutathione agarose, followed by the incubation with cell lysates of POU1F1-overexpressing HEK293T or SGC7901 cells. The immobilized proteins were then eluted and subjected to western blot analysis.

### Co-immunoprecipitation (Co-IP)

Cells were co-transfected with V5-tagged POU1F1 and HA-tagged HMGA1B/HA-tagged HMGA2. Cells were harvested 48 h post-transfection. Protein lysates were prepared in IP lysis buffer supplemented with Pierce protease inhibitor cocktail (Pierce). Co-IP was performed using Pierce Crosslink IP Kit (Pierce) according to the manufacture’s protocols. In brief, 1000 μg cell lysates were incubated with anti-V5 or anti-HA conjugated agarose. The total cell lysates served as input control. IgG (Abcam) was used as a control. The immunocomplexes were eluted and subjected to western blot analysis.

### Chromatin immunoprecipitation (ChIP) assay

ChIP assay was conducted using Pierce Agarose ChIP Kit (Pierce) according to the manufacturer’s instructions. In brief, BGC823 and SGC7901 cells were crosslinked with 1% formaldehyde, followed by MNase digestion. The digested chromatin was incubated with anti-HMGA1 (Abcam), anti-HMGA2 (GeneTex, USA), or normal IgG for immunoprecipitation. DNA was then purified and analyzed by qRT-PCR assay.

### Dual luciferase reporter assay

The promoter region p(−1321/+15) of POU1F1 was amplified by PCR and cloned into pGL-3 Basic vector (Promega, Madison, WI, USA) as described^24^. BGC823 and SGC7901 cells were co-transfected with POU1F1-p(−1321/+15) Luc and HMGA1B/HMGA2 overexpression construct using Lipofectamine 2000 transfection reagent (Invitrogen). Dual luciferase reporter assay was performed using Dual Luciferase Reporter Assay System (Promega). Renilla luciferase activity was used to normalize transfection efficiency.

### Flow cytometry

THP-1 cells were differentiated using 200 nM 12-myristate 13-acetate (PMA, Sigma–Aldrich) as previously described^25^. FACS was used for the detection of CD14, CD11b, F4/80, and CD11c. Briefly, macrophages were harvested and stained with FITC-CD14, PE-CD11b, PE-F4/80, and FITC-CD11c antibodies (BD Biosciences), followed by four-color FACS analysis with FACSCailbur (BD Biosciences). All data were analyzed by FlowJo software (Tree Star, Ashland, OR, USA).

### Statistical analysis

All results were presented as means ± standard deviation (SD). All experiments were performed at least three times. Statistical analysis was performed using Student’s *t*-test between two groups. For multiple comparison, one-way ANOVA followed by Tukey’s multiple comparison test was performed. Statistical analysis was conducted using the SPSS22.0 (SPSS Inc., Chicago, IL, USA). Differences were considered statistically significant when *P < 0.05. **P < 0.01.

## Results

### Upregulated POU1F1 is associated with poor prognosis in GC

**Fig. 1:**
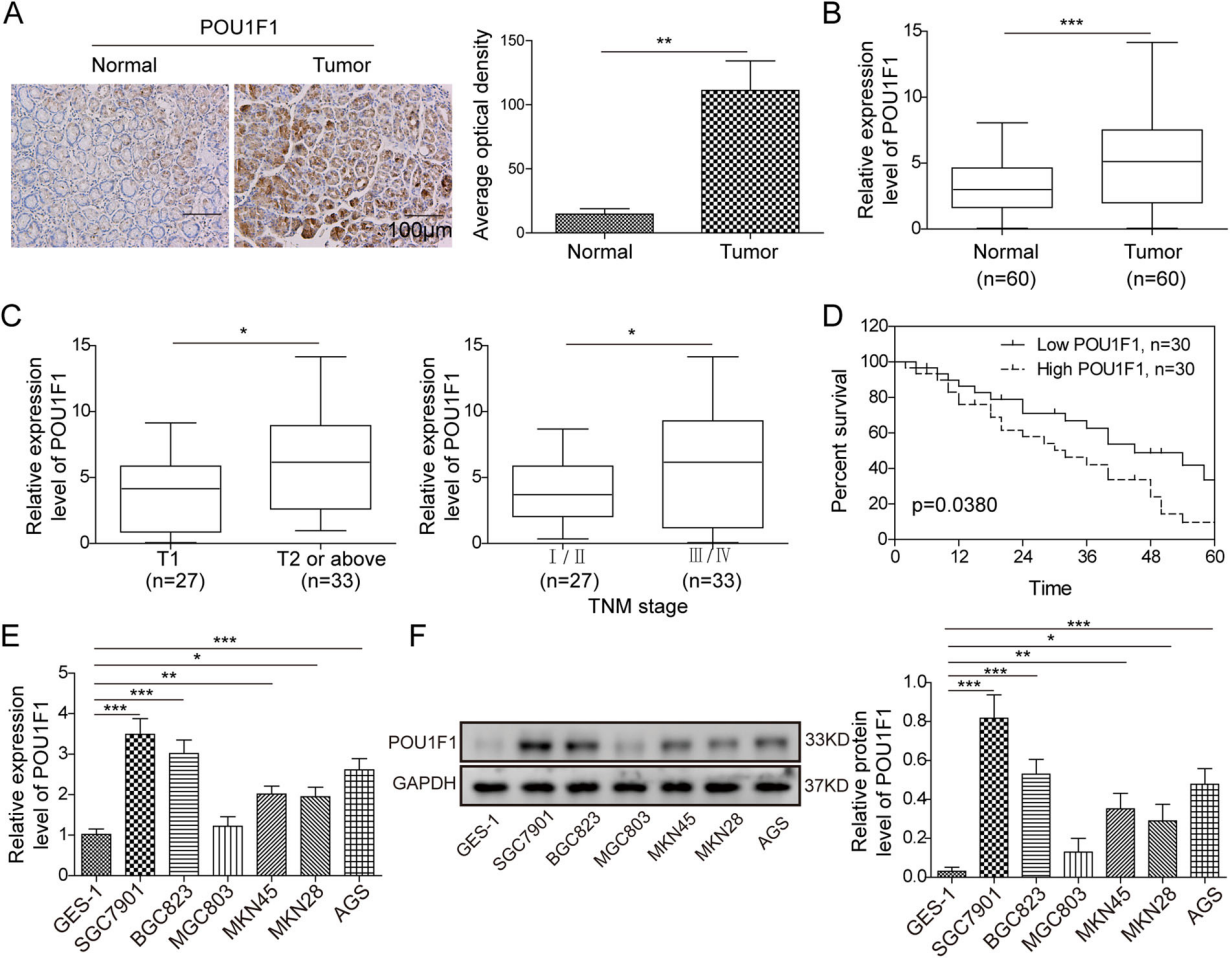
Upregulated POU1F1 is associated with poor prognosis in GC. **A** Expression of POU1F1 in GC and paired adjacent normal tissues were determined by IHC. **B** The mRNA level of POU1F1 in GC and paired adjacent normal tissues were determined by qRT-PCR assay. **C** Expression patterns of POU1F1 in T1 or T2/above and different TNM stages were determined by qRT-PCR analysis. **D** Kaplan–Meier curves for overall survival of GC patients. **E,** **F** The mRNA and protein levels of POU1F1 in GC cell lines and GES-1 cells were examined using qRT-PCR and western blot analysis, respectively. GAPDH served as an internal control. Data were representative images or were expressed as the mean ± SD of *n* = 3 experiments. *P < 0.05, **P < 0.01, ***P < 0.001.

To explore the biological function of POU1F1, we first examined the expression of POU1F1 in GC. As shown in Fig. 1A, POU1F1 was highly expressed in GC tissues compared to paired adjacent normal tissues as detected by IHC analysis. The qRT-PCR results confirmed the elevated expression of POU1F1 in GC specimens (Fig. 1B). Moreover, the correlation between POU1F1 expression and the clinical-pathological factors of GC patients was determined and the data presented that POU1F1 expression was positively with invasion depth and TNM stage (Fig. 1C), which means the higher POU1F1 expression, the deeper invasion depth or the advanced TNM stage. In addition, the overall survival rates of patients with high POU1F1 expression were significantly poorer than that of patients with low POU1F1 expression (Fig. 1D), suggesting that POU1F1 expression served as a prognostic factor in GC. Furthermore, we next examined POU1F1 expression in six different GC cell lines. Consistently, the mRNA and protein levels of POU1F1 were remarkably upregulated in GC cell lines including SGC7901, BGC823, MGC803, MKN45, MKN28, and AGS cells, in comparison with gastric epithelial cell line GES-1 cells (Fig. 1E, F). Given the relatively high expression of POU1F1 in SGC7901 and BGC823 cells, these two cell lines were thus selected for subsequent experiments. Taken together, these findings indicated that upregulated POU1F1 is associated with poor prognosis in GC.

### Knockdown of POU1F1 inhibits cell proliferation, migration, invasion, and angiogenesis in GC cells

**Fig. 2:**
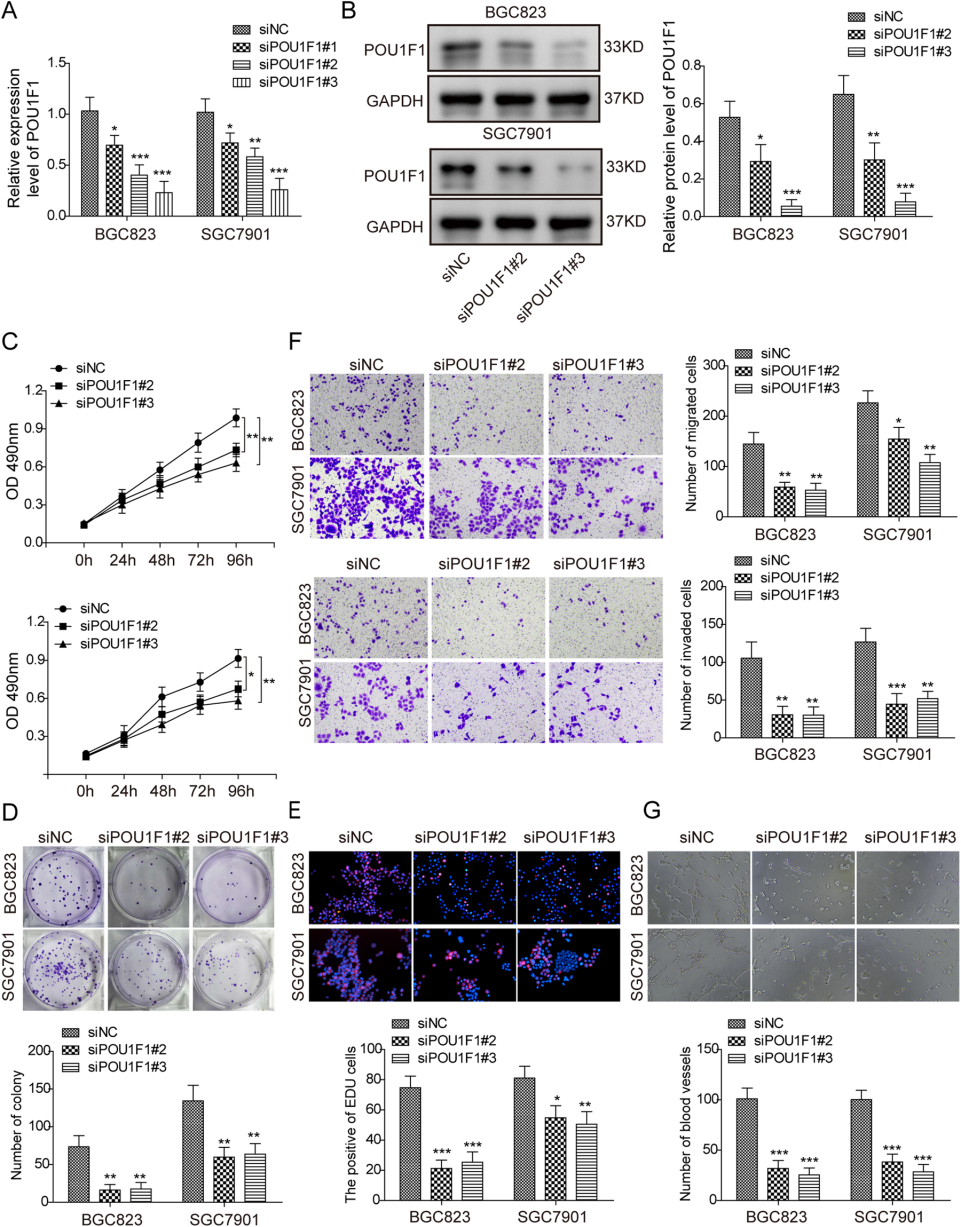
Knockdown of POU1F1 inhibits cell proliferation, migration, invasion, and angiogenesis in GC cells. **A** The mRNA level of POU1F1 was determined by qRT-PCR analysis. **B** The protein level of POU1F1 was determined by western blot. GAPDH served as a loading control. **C** Cell proliferation was monitored by CCK-8 assay. **D** Colony forming ability was assessed by colony formation assay. **E** DNA synthesis and cell proliferation was monitored by EdU incorporation assay. Red, EdU; Blue, DAPI. **F** The capacity of cell migration and invasion were assessed by transwell system. **G** In vitro angiogenesis was monitored by tube formation assay. Data were representative images or were expressed as the mean ± SD of *n* = 3 experiments. *P < 0.05, **P < 0.01, ***P < 0.001.

Knockdown experiments were further conducted to investigate the functional role of POU1F1 in GC cells. In BGC823 and SGC7901 cells, si-POU1F1 #1 was found to be less effective in silencing POU1F1 based on qRT-PCR results (Fig. 2A), thus it was not used for subsequent investigations. si-POU1F1 #2 and si-POU1F1 #3 significantly decreased POU1F1 mRNA and protein levels in both BGC823 and SGC7901 cells (Fig. 2A, B). CCK-8 and colony formation assays revealed that knockdown of POU1F1 notably inhibited cell proliferation and suppressed the colony forming abilities of BGC823 and SGC7901 cells, respectively (Fig. 2C, D). The EdU incorporation rates were also markedly decreased by si-POU1F1 #2 and si-POU1F1 #3 in both BGC823 and SGC7901 cells (Fig. 2e), indicating that the proportion of proliferating cells were significantly reduced in POU1F1-knockdown cells. In addition, transwell assay showed that silencing of POU1F1 dramatically inhibited the migratory and invasive capacities of BGC823 and SGC7901 cells (Fig. 2F). In vitro angiogenesis was also inhibited in POU1F1-knockdown cells in which the number, length and area of capillary-like structures were significantly decreased (Fig. 2G). In contrast, overexpression of POU1F1 exhibited opposite effects on cell proliferation, migration, invasion, and angiogenesis in BGC823 and SGC7901 cells (Fig. S1a-d). Collectively, these data suggest an oncogenic role of POU1F1 in the tumorigenesis of gastric cancer.

### Knockdown of POU1F1 inhibits tumor growth and metastasis in vivo

**Fig. 3:**
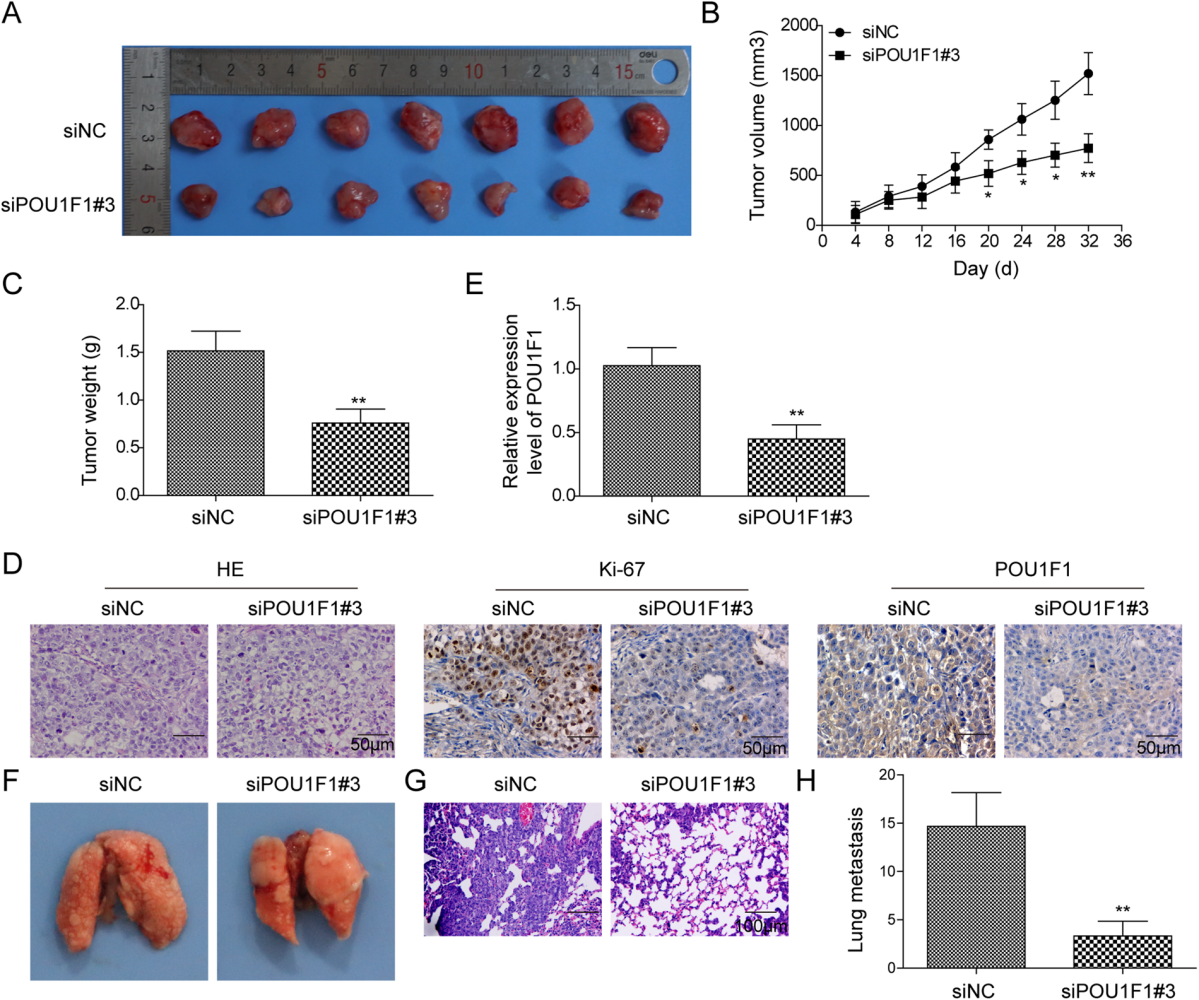
Knockdown of POU1F1 inhibits tumor growth and metastasis in vivo. **A** The photos of xenograft tumors. **B** Quantitative analysis of tumor size. **C** Quantitative analysis of tumor weight. **D** Histopathological analysis of xenograft tumors. The histopathological changes were determined by H&E staining. The immunoreactivities of POU1F1 and Ki67 were assessed by IHC analysis. **E** The mRNA level of POU1F1 in xenograft tumors were determined by qRT-PCR. GAPDH served as an internal control. **F** The photos of lung tissues. **G** Histopathological analysis of metastatic nodules in lung. The histopathological changes were determined by H&E staining. **H** Quantitative analysis of metastatic nodule numbers. Data were representative images or were expressed as the mean ± SD of *n* = 3 experiments. *P < 0.05, **P < 0.01.

In order to validate the oncogenic role of POU1F1 in vivo, xenograft mouse models were generated. Given si-POU1F1 #3 resulting in more efficient POU1F1 silencing, it was thus selected for the in vivo study. As shown in Fig. 3A, B, knockdown of POU1F1 significantly decreased the tumor size compared with that of control group. si-POU1F1 #3 resulted in a dramatic reduction of tumor weight after subcutaneous inoculation (Fig. 3C). Xenograft tumors were then subjected to histopathological analysis. As expected, silencing of POU1F1 remarkably decreased POU1F1-positive expression in xenograft tumors, as well as the cell proliferation marker Ki67 (Fig. 3D), which was also certified by qRT-PCR analysis (Fig. 3E). Additionally, we further explored the effect of POU1F1 on lung metastasis in vivo. POU1F1-knockdown or control cells were injected into the tail veins of mice. As presented in Fig. 3F-H, the observed metastatic lung nodules were significantly decreased in si-POU1F1 #3 group, compared with that of control mice. Together, these findings indicate the oncogenic role of POU1F1 in tumor growth and metastasis in vivo.

### HMGA1B/2 transcriptionally activates POU1F1

**Fig. 4:**
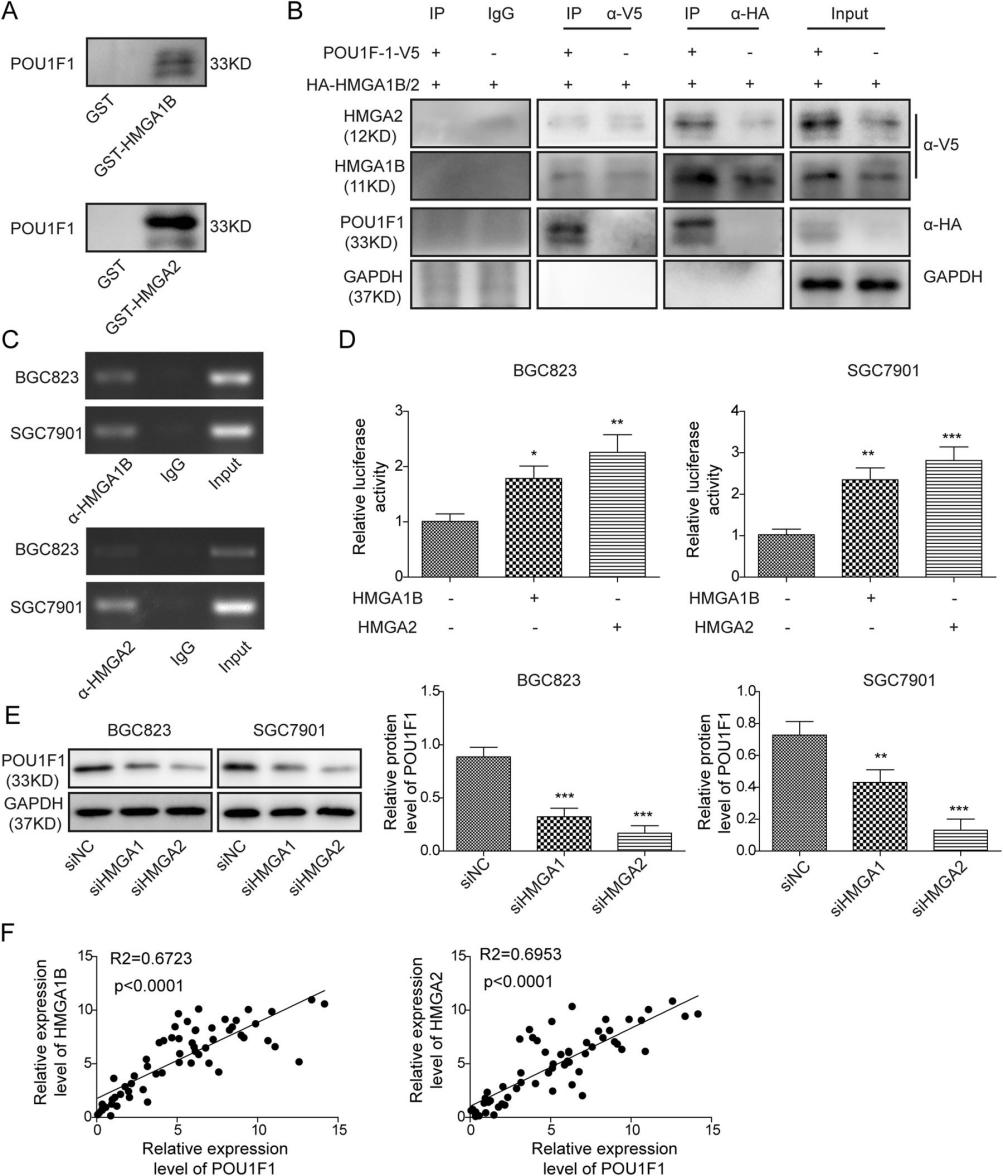
HMGA1B/2 upregulates POU1F1 transcriptionally. **A** In vitro interactions between POU1F1 and HMGA1B or HMGA2 were determined by GST pull-down assay. **B** In vivo interactions between POU1F1 and HMGA1B or HMGA2 were determined by co-IP. Whole-cell lysates served as an input control. **C** The enrichment of HMGA1 or HMGA2 at POU1F1 promoter was assessed by ChIP assay. Normal IgG served as a negative control. The non-immunoprecipitated chromatin served as an input control. **D** POU1F1 promoter region containing sequence between nt −1321 and +15 was cloned into pGL-3 Basic vector. Luciferase activity was determined by dual luciferase reporter assay. Renilla luciferase activity served as an internal control. **E** The protein level of POU1F1 was determined by western blot. GAPDH served as a loading control. **F** The correlations between POU1F1 and HMGA1B or HMGA2 in GC were determined by Pearson correlation analysis. Data were expressed as the mean ± SD of *n* = 3 experiments. *P < 0.05, **P < 0.01, ***P < 0.001.

Previous study has demonstrated that POU1F1 was upregulated by HMGA proteins in pituitary tumors^24^. Considering the significant upregulation of HMGA1 and HMGA2 in GC^26^, we next tested whether HMGA proteins were involved in the regulation of POU1F1 in GC cells. GST pull-down assay revealed that GST-HMGA1B or GST-HMGA2 successfully pulled down POU1F1 recombinant protein in HEK293T cells (Fig. 4A). Similar results were also found in SGC7901 cells (Fig. S3). To validate this result, HEK293T cells were co-transfected with V5-POU1F1 and HA-HMGA1B/2, followed by Co-IP analysis. As shown in Fig. 4B, anti-V5 antibody immunoprecipitated HA-HMGA1B and HA-HMGA2 when compared with corresponding control. Conversely, anti-HA antibody also successfully immunoprecipitated V5-POU1F1, and the interactions between POU1F1 and HMGA1B/2 were not interrupted by ethidium bromide (EtBr) (Fig. 4B), suggesting that the bindings between POU1F1 and HMGA1B/2 were DNA-independent protein association. It is worthy to note that co-transfection of V5-POU1F1 and HA-HMGA1B/2 remarkably induced HMGA1B and HMGA2 expression compared to HA-HMGA1B/2 alone group (Fig. 4B), indicating that POU1F1 also upregulated HMGA1B and HMGA2 expression. In order to investigate whether HMGA proteins modulated POU1F1 via transcriptional regulation, ChIP and dual luciferase reporter assays were conducted. ChIP assays revealed that HMGA1B and HMGA2 were significantly enriched at POU1F1 promoter region in both BGC823 and SGC7901 cells (Fig. 4C). Co-transfection of POU1F1-p(−1321/+15) Luc and HMGA1B or HMGA2 overexpression construct led to a remarkable induction of promoter activity in BGC823 and SGC7901 cells (Fig. 4D), indicating that HMGA1B or HMGA2 positively regulated POU1F1 expression at the transcriptional level. Consistently, knockdown of HMGA1 or HMGA2 caused a significant reduction of POU1F1 in BGC823 and SGC7901 cells (Fig. 4E). In addition, the Pearson correlation analysis showed that POU1F1 and HMGA1 or HMGA2 were positively correlated in GC tissues (Fig. 4F). These findings suggest that HMGA1B or HMGA2 positively regulates POU1F1 at the transcriptional level.

### POU1F1 promotes GC progression via regulating macrophage proliferation, migration, polarization, and angiogenesis

**Fig. 5:**
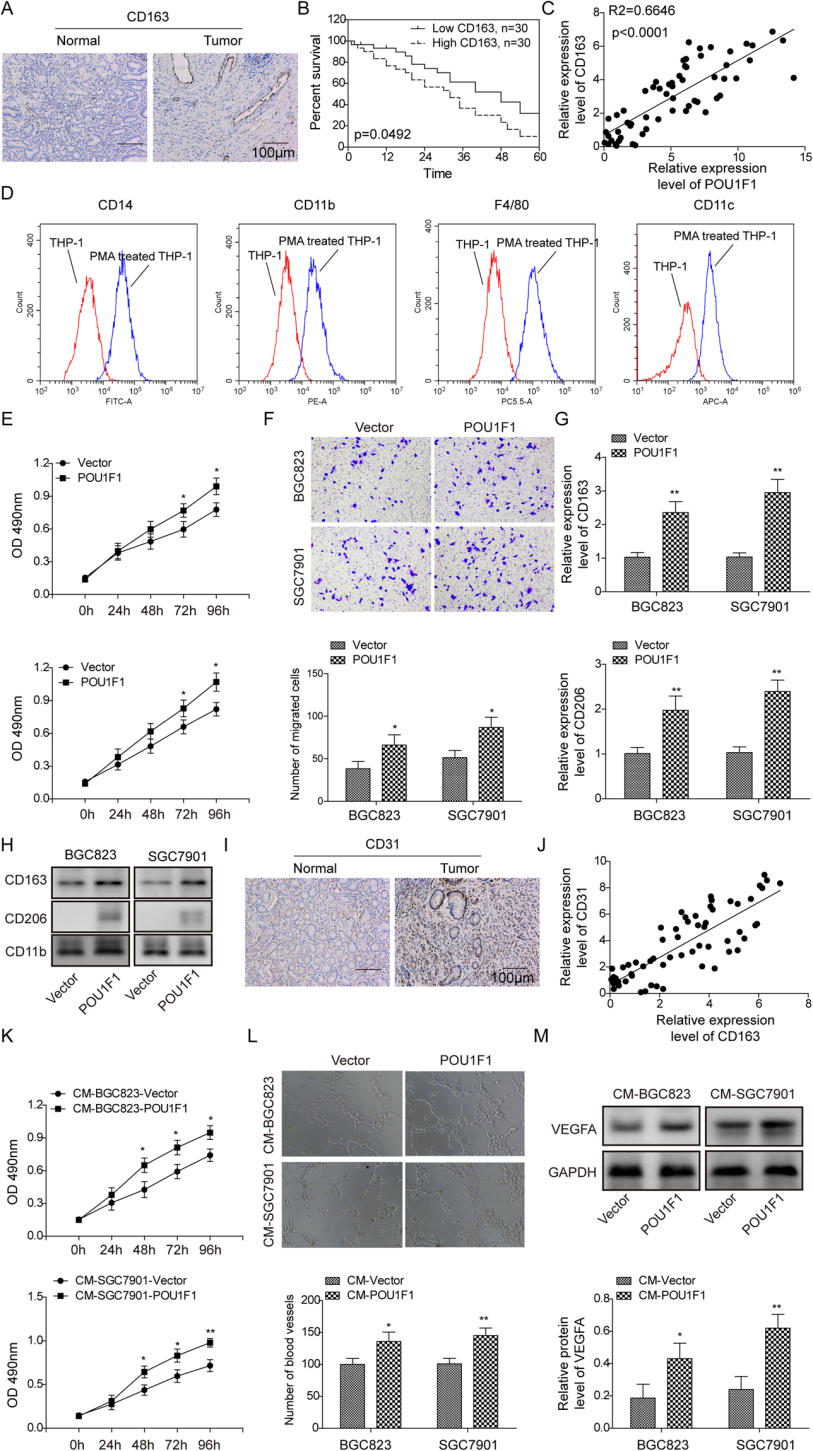
POU1F1 promotes GC progression via regulating macrophage proliferation, migration, polarization, and angiogenesis. **A** The immunoreactivity of CD163 was assessed by IHC analysis. **B** Kaplan–Meier curves for overall survival of GC patients. **C** The correlation between CD163 and POU1F1 in GC was determined by Pearson correlation analysis. **D** Cell surface CD14, CD11b, F4/80, and CD11c expression were analyzed by flow cytometry. **E** Cell viability of macrophages was monitored by CCK-8 assay. **F** Cell migration of macrophages was determined by transwell migration assay. The mRNA (**G**) and protein (**H**) levels of CD163 and CD206 were detected by qRT-PCR and western blot, respectively. CD11b served as an internal control. (**I**) The immunoreactivity of CD31 was assessed by IHC analysis. **J** The correlations between CD31 and CD163 in GC were determined by Pearson correlation analysis. **K** Cell viability of HUVECs was monitored by CCK-8 assay. **L** In vitro angiogenesis was monitored by tube formation assay. **M** The protein level of VEGF was determined by western blot. GAPDH served as a loading control. Data were representative images or were expressed as the mean ± SD of *n* = 3 experiments. *P < 0.05, **P < 0.01.

In order to investigate the relation between POU1F1 and macrophages, we next examined the expression of M2 phenotype marker CD163 in GC tissues. As shown in Fig. 5A, the immunoreactivity of CD163 was distinctly stronger in GC tissues compared to their normal counterparts. The patients with high CD163 expression exhibited poor overall survival, compared with patients expressing low levels of CD163 (Fig. 5B). In addition, there was a significant positive correlation between POU1F1 and CD163 (Fig. 5C). PMA-stimulated macrophage differentiation of THP-1 monocytes is a widely used model to study monocyte-macrophage polarization^25^. Flow cytometry analysis showed that the surface levels of macrophage markers CD14, CD11b, F4/80, and CD11c were significantly increased upon PMA treatment (Fig. 5D), indicating that THP-1 cells differentiated into macrophage-like cells. These cells were then co-cultured with control or POU1F1-overexpressing GC cells. CCK-8 assays revealed that overexpression of POU1F1 accelerated macrophage growth in comparison with control group (Fig. 5E). The migratory capacities of macrophages co-cultured with POU1F1-overexpressing GC cells were significantly increased as assessed by transwell migration assay (Fig. 5F). Moreover, macrophages co-cultured with POU1F1-overexpressing GC cells exhibited markedly increased CD163 and CD206 expression, which were well-known M2 macrophage markers (Fig. 5G, H). It is well-accepted that M2-like macrophages play an important role in angiogenesis^10^. To delineate the proangiogenic effect of POU1F1 overproduced by GC cells on macrophages, the endothelial marker CD31 was examined by IHC analysis. As presented in Fig. 5I, CD31 was significantly upregulated in GC tissues compared with paired adjacent normal gastric tissues. Pearson correlation analysis indicated that CD31 positively correlated with CD163 in GC tissues (Fig. 5J). Furthermore, we next validated these results in vitro. HUVECs were cultured in the conditioned medium (CM) from co-culture of GC cells and macrophages. The cell proliferation and angiogenesis of HUVECs cultured with CM-BGC823-POU1F1 or CM-SGC7901-POU1F1 were remarkably increased compared to corresponding controls (Fig. 5K, L). Consistent with the results of tube formation assay, the angiogenesis marker VEGFA was dramatically upregulated in HUVECs cultured with CM-BGC823-POU1F1 or CM-SGC7901-POU1F1 (Fig. 5M). Taken together, these data indicate that POU1F1 promotes GC progression via regulating macrophage proliferation, migration, polarization, and angiogenesis.

### CXCL12/CXCR4 axis is involved in POU1F1-induced macrophage polarization

**Fig. 6:**
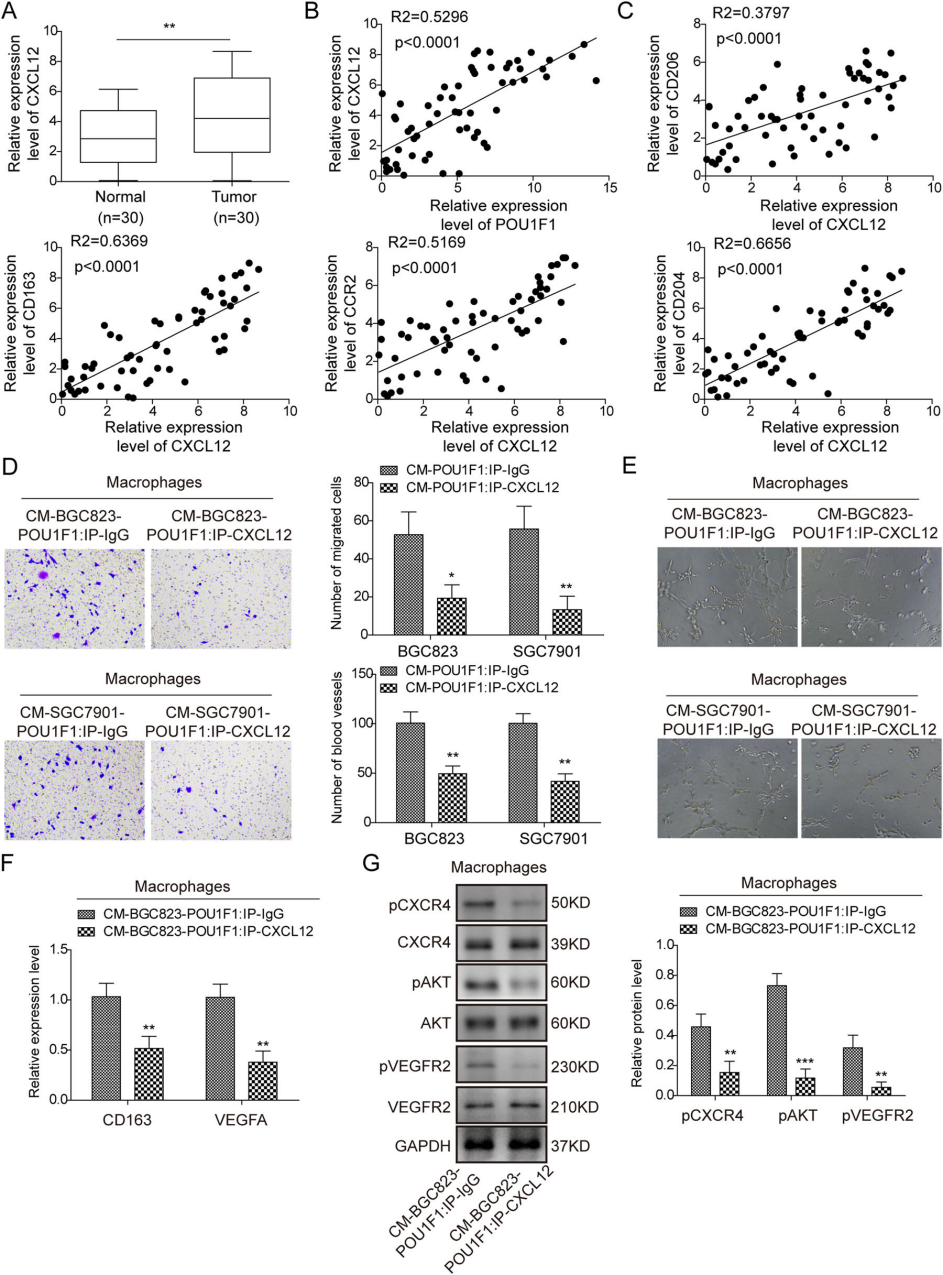
CXCL12/CXCR4 axis is involved in POU1F1-induced macrophage polarization. **A** The mRNA level of CXCL12 was determined by qRT-PCR. GAPDH served as an internal control. **B** The correlation between CXCL12 and POU1F1 in GC was determined by Pearson correlation analysis. **C** The correlations between CXCL12 and CD206, CD163, CCR2, or CD204 in GC were determined by Pearson correlation analysis. **D** Cell migration of macrophages was determined by transwell migration assay. **E** In vitro angiogenesis was monitored by tube formation assay. **F** The mRNA levels of CD163 and VEGFA were determined by qRT-PCR. GAPDH served as an internal control. **G** The protein levels of p-CXCR4, CXCR4, p-Akt, Akt, p-VEGFR2, and VEGFR2 were determined by western blot. GAPDH served as a loading control. Data were representative images or were expressed as the mean ± SD of *n* = 3 experiments. *P < 0.05, **P < 0.01, ***P < 0.001.

Previous studies have demonstrated that POU1F1/CXCL12/CXCR4 axis contributes to macrophage recruitment and polarization, thereby promoting breast cancer metastasis^14,15^. To further unravel the underlying mechanism by which POU1F1 promoted metastasis in GC, we next examined the expression of CXCL12 in GC tissues. As shown in Fig. 6A, elevated expression of CXCL12 was observed in GC tissues, compared with normal gastric tissues. Pearson correlation analysis revealed that CXCL12 positively correlated with POU1F1 in GC tissues (Fig. 6B), and CXCL12 also positively correlated with M2 phenotype markers CD206, CD163, CCR2, and CD204 (Fig. 6C). Additionally, ELISA assay revealed that CXCL12 was markedly upregulated in CM-BGC823-POU1F1 or CM-SGC7901-POU1F1 compared to corresponding controls (Fig. S2a), indicating that CXCL12 might be a key contributor to POU1F1-induced macrophage polarization. To test this hypothesis, CM-BGC823-POU1F1 or CM-SGC7901-POU1F1 was immunoprecipitated with either anti-CXCL2 antibody (CM-BGC823-POU1F1 IP: CXCL12 or CM-SGC7901-POU1F1 IP: CXCL12) or normal IgG (CM-BGC823-POU1F1 IP: IgG or CM-SGC7901-POU1F1 IP: IgG). Macrophages were then maintained in the immunoprecipitated CM for 24 h. Transwell migration assay showed that macrophages cultured with CM-BGC823-POU1F1 IP: CXCL12 or CM-SGC7901-POU1F1 IP: CXCL12 exhibited decreased migratory capacities in comparison with corresponding control cells (Fig. 6D). Interestingly, the elevation of VEGFA was found in CM-BGC823-POU1F1 or CM-SGC7901-POU1F1 (Fig. S2b), suggesting the proangiogenic role of POU1F1. Figure 6E showed a significant impairment of angiogenesis in HUVECs cultured with CM-BGC823-POU1F1 IP: CXCL12 or CM-SGC7901-POU1F1 IP: CXCL12. In addition, a remarkable reduction of CD163 and VEGFA mRNA levels were found in macrophages cultured with CM-BGC823-POU1F1 IP: CXCL12 or CM-SGC7901-POU1F1 IP: CXCL12 (Fig. 6F). Furthermore, the impairments of cell migration and angiogenesis were accompanied with decreased p-CXCR4, p-Akt, and p-VEGFR2 expression, confirming that CXCL12/CXCR4 axis, p-Akt and p-VEGFR2 were involved in this process (Fig. 6G). Together, these findings suggest that CXCL12/CXCR4 axis plays an important role in POU1F1-induced macrophage polarization.

### POU1F1 promotes GC metastasis in lung by modulating macrophage polarization through CXCL12/CXCR4 axis

**Fig. 7:**
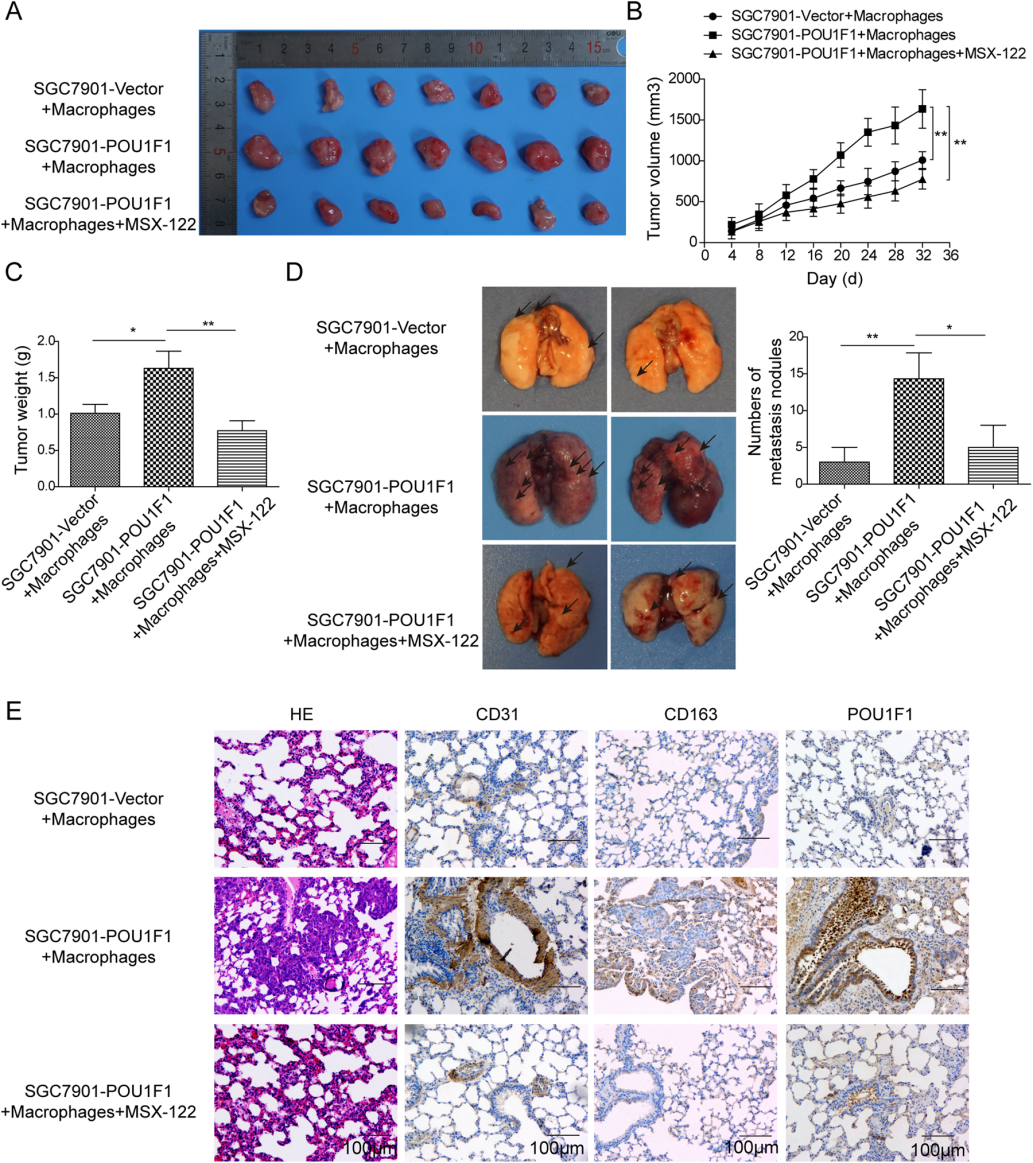
POU1F1 promotes GC metastasis in lung by modulating macrophage polarization through CXCL12/CXCR4 axis. **A** The photos of xenograft tumors. **B** Quantitative analysis of tumor size. **C** Quantitative analysis of tumor weight. **D** The photos of lung tissues. Quantitative analysis of metastatic nodule numbers. **E** Histopathological analysis of metastatic nodules in lung. The histopathological changes were determined by H&E staining. The immunoreactivities of CD31, CD163, and POU1F1 were assessed by IHC analysis.

In order to assess the critical role of CXCL12/CXCR4 axis in vivo, POU1F1-overexpressing SGC7901 or control cells and macrophages were co-injected to nude mice. The CXCR4 antagonist MSX-122 was employed to block the effect of CXCR4. As shown in Fig. 7A, B, tumors derived from SGC7901-POU1F1+ macrophages grew faster than that derived from control cells, while this effect was abrogated by MSX-122. Tumors were harvested 7 weeks after inoculation, and the tumor weights in SGC7901-POU1F1+ macrophages group were significantly increased compared with control group. Upon MSX-122 treatment, the tumor weights were markedly decreased in comparison with SGC7901-POU1F1+ macrophages group (Fig. 7C). Moreover, we further examined the metastasis in lung. The numbers of metastatic nodules were dramatically increased in SGC7901-POU1F1+ macrophages group, whereas MSX-122 abolished this effect on metastasis (Fig. 7D). Consistent with this observation, metastatic nodules were detected in H&E-stained lung tissues, and metastatic nodules were hardly observed in the presence of MSX-122 (Fig. 7E). It is worthy to note that CD31, CD163, and POU1F1 were significantly upregulated in xenograft tumors derived from SGC7901-POU1F1+ macrophages, whereas upregulation of these proteins were reversed by MSX-122 as assessed by IHC analysis (Fig. 7E). These findings indicate that POU1F1 promotes GC metastasis in lung by modulating macrophage polarization through CXCL12/CXCR4 axis.

## Discussion

In the present study, we reported that POU1F1 was significantly upregulated in GC tissues and cells, and associated with poor prognosis in GC. Both in vitro and in vivo experiments revealed the oncogenic role of POU1F1 in GC, and it was positively regulated by HMGA1B/2 at the transcriptional level. Moreover, macrophage proliferation, migration, polarization, and angiogenesis played important roles in POU1F1-mediated GC progression, and this process relied on CXCL12/CXCR4 axis. Subsequent in vivo experiments confirmed that POU1F1 promoted GC metastasis in lung by modulating macrophage polarization through CXCL12/CXCR4 axis.

Accumulating evidence has elucidated the significance of tumor microenvironment in prognosis prediction and therapeutic efficacy^27,28^. Interactions between cancer cells and the microenvironment are mediated by autocrine or paracrine cytokines, chemokines, growth factors and ECM-remodeling molecules^4^. As the most abundant immune cells in the tumor microenvironment, macrophages, TAMs in particular, have shown a great potential as biomarker for GC^5^. In this study, we observed significantly elevated expression of M2 phenotype marker CD163 in GC tissues, and GC patients with high CD163 expression exhibited poor overall survival rate. These findings promoted us to speculate that macrophage polarization might play critical roles in GC progression.

POU1F1, which belongs to POU family, is responsible for pituitary development and transcriptional activation of pituitary genes, such as prolactin (PRL), growth hormone (GH), and POU1F1 itself. In recent years, several studies support a critical role for POU1F1 in breast cancer progression and metastasis^13–15^. A more recent study has demonstrated that POU1F1 promotes breast cancer metastasis via recruitment and polarization of macrophages into TAMs^15^. Our data showed that POU1F1 was also upregulated in GC tissues and cells, and the aberrant expression of POU1F1 promotes cell proliferation, migration, and angiogenesis. Interestingly, POU1F1 also positively correlated with M2 phenotype marker CD163 in GC tissues, raising the possibility that POU1F1 might exert oncogenic role via modulating macrophage polarization. Subsequent experiments showed that overexpression of POU1F1 in GC remarkably increased M2 phenotype markers CD163 and CD206 expression in macrophages, indicating the critical role of POU1F1 in macrophage polarization. In accordance with the findings in breast cancer^14,15^, chemokine CXCL12 was also found as an important mediator in POU1F1-induced macrophage polarization in GC. It has been demonstrated that CXCL12 and its receptor CXCR4 are involved in cancer cell proliferation, migration, invasion, and angiogenesis^29^. More importantly, CXCL12 also contributes to polarization of macrophages into TAMs^30^. Upon CXCL12 binding, CXCR4 activates a variety of signaling cascades, including Akt signaling pathway^29^. In the current study, we found that lack of CXCL12 in CM significantly inhibited phosphorylation of CXCR4, Akt, and VEGFR2. Our data also revealed a paracrine mechanism by which CXCL12 secreted by GC cells bound to endothelial CXCR4 to promote angiogenesis in HUVECs. By using the CXCR4 antagonist MSX-122, the oncogenic role of CXCL12/CXCR4 was also validated in vivo.

In addition, we also reported that HMGA1B and HMGA2 served as upstream regulators of POU1F1 in GC. GST pull-down assay and co-IP unequivocally demonstrated that HMGA1B or HMGA2 interacted with POU1F1 directly. Further ChIP and luciferase reporter assays confirmed that the promoter region nt −1321/+15 of POU1F1 was responsible for the HMGA1B/2 binding. Both HMGA1B or HMGA2 positively regulated POU1F1 in GC cells, this is consistent with a previous report, which illustrated that HMGA proteins upregulated POU1F1 in pituitary cancer^24^. Emerging evidence suggest that *HMGA* function as oncogenes in GC^18,20,21,23^. Our findings provided a novel mechanism by which HMGA proteins promotes GC progression.

In conclusion, we have demonstrated that HMGA1B/2 transcriptionally upregulated POU1F1 in GC. Elevated POU1F1 promoted GC metastasis via regulating macrophage polarization in a CXCL12/CXCR4-dependent manner.

## Supplementary information

Supplementary figure legends

FIGS1

FIGS2

FIGS3
