# Supplementary material for: HMGA1B/2 transcriptionally activated-POU1F1 facilitates gastric carcinoma metastasis via CXCL12/CXCR4 axis-mediated macrophage polarization
Source: Cell Death Dis. 2021 Apr 29;12(5):422. doi: 10.1038/s41419-021-03703-x (PMC8084942; doi:10.1038/s41419-021-03703-x)
Supplement: Supplementary file 1 — Supplementary figure legends [file 41419_2021_3703_MOESM1_ESM.docx]

**Supplementary figure legends**

**
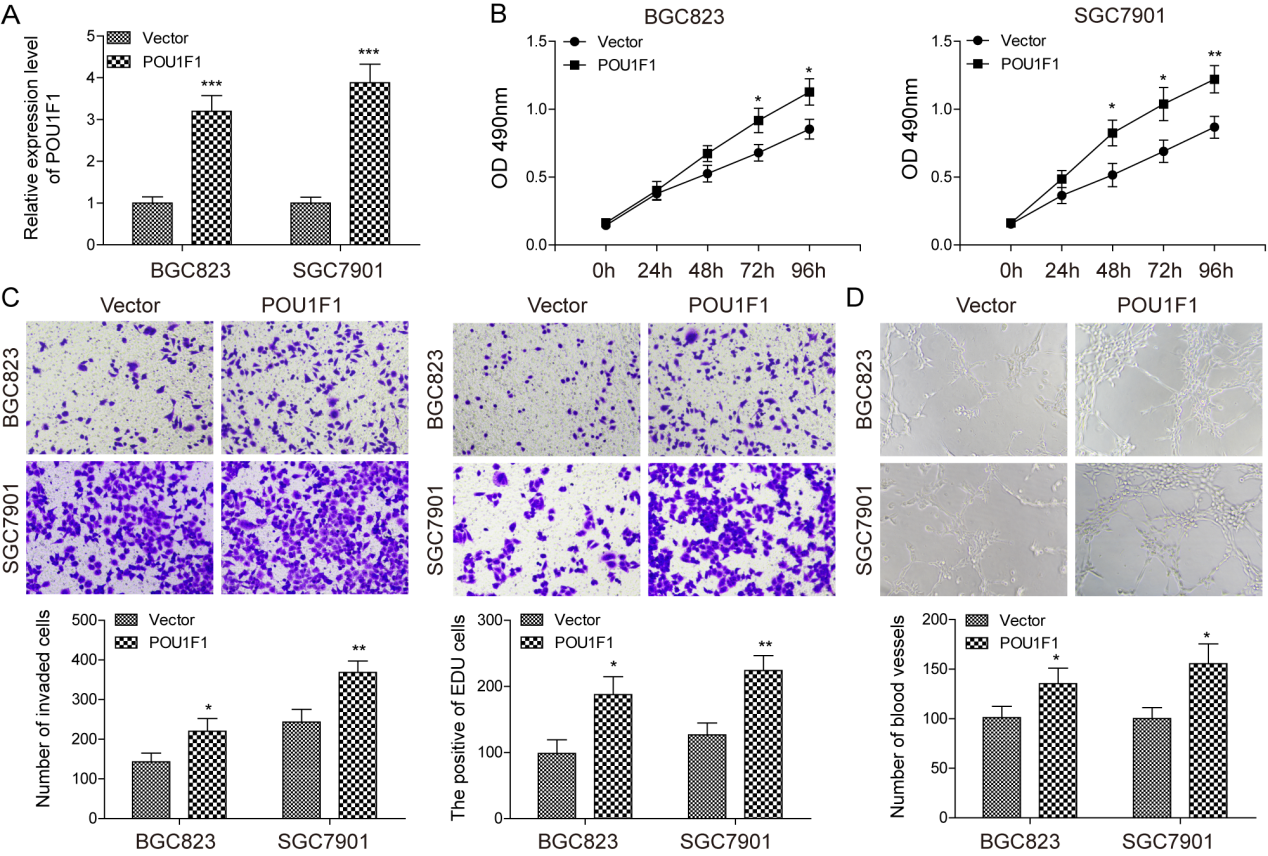
**

**Figure S1. Overexpression of POU1F1 promotes cell proliferation, migration, invasion and angiogenesis in GC cells.** (A) The mRNA level of POU1F1 was determined by qRT-PCR analysis. (B) Cell proliferation was monitored by CCK-8 assay. (C) The capacity of cell migration and invasion were assessed by transwell system. (D) *In vitro* angiogenesis was monitored by tube formation assay. Data were representative images or were expressed as the mean ± SD of *n = 3* experiments. *, P < 0.05, **, P < 0.01, ***, P<0.001.


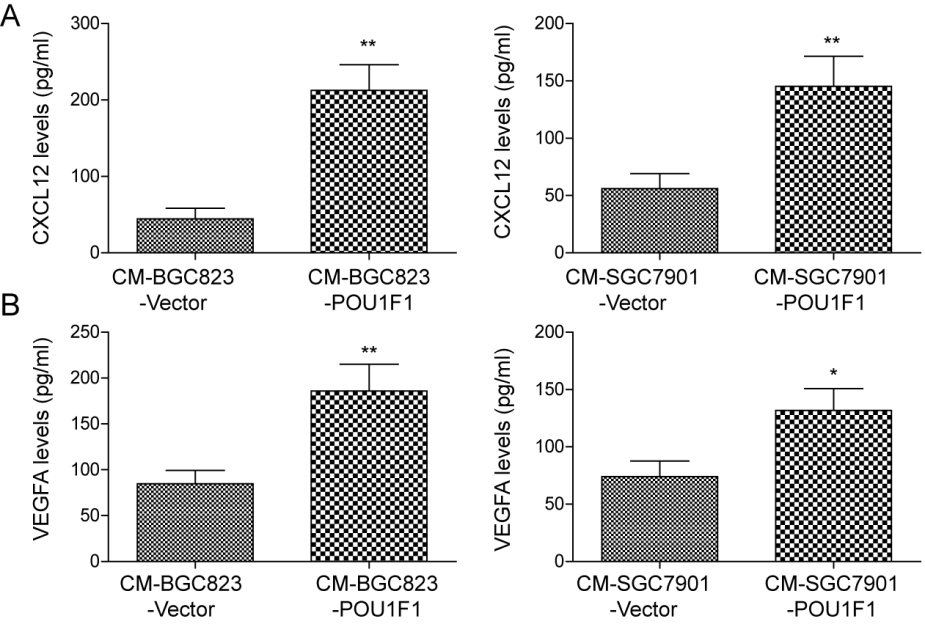


**Figure S2. Overexpression of POU1F1 induces the levels of CXCL12 and VEGFA in CM.** The levels of CXCL12 (A) and VEGFA (B) in CM were detected by ELISA assays. Data were expressed as the mean ± SD of *n = 3* experiments. *, P < 0.05, **, P < 0.01.


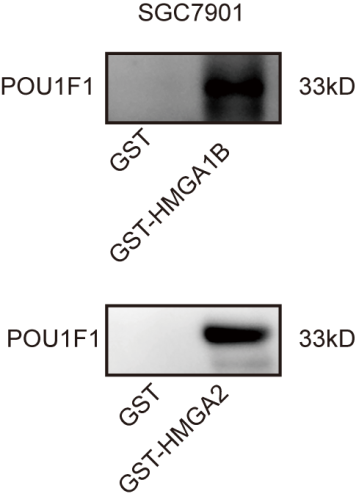


**Figure S3. HMGA1B/2 interacts with POU1F1 in SGC7901 cells.** *In vitro* interactions between POU1F1 and HMGA1B or HMGA2 were determined by GST pull-down assay in SGC7901 cells.
