# Supplementary figures and images for: HMGA1B/2 transcriptionally activated-POU1F1 facilitates gastric carcinoma metastasis via CXCL12/CXCR4 axis-mediated macrophage polarization
Source: Cell Death Dis. 2021 Apr 29;12(5):422. doi: 10.1038/s41419-021-03703-x (PMC8084942; doi:10.1038/s41419-021-03703-x)

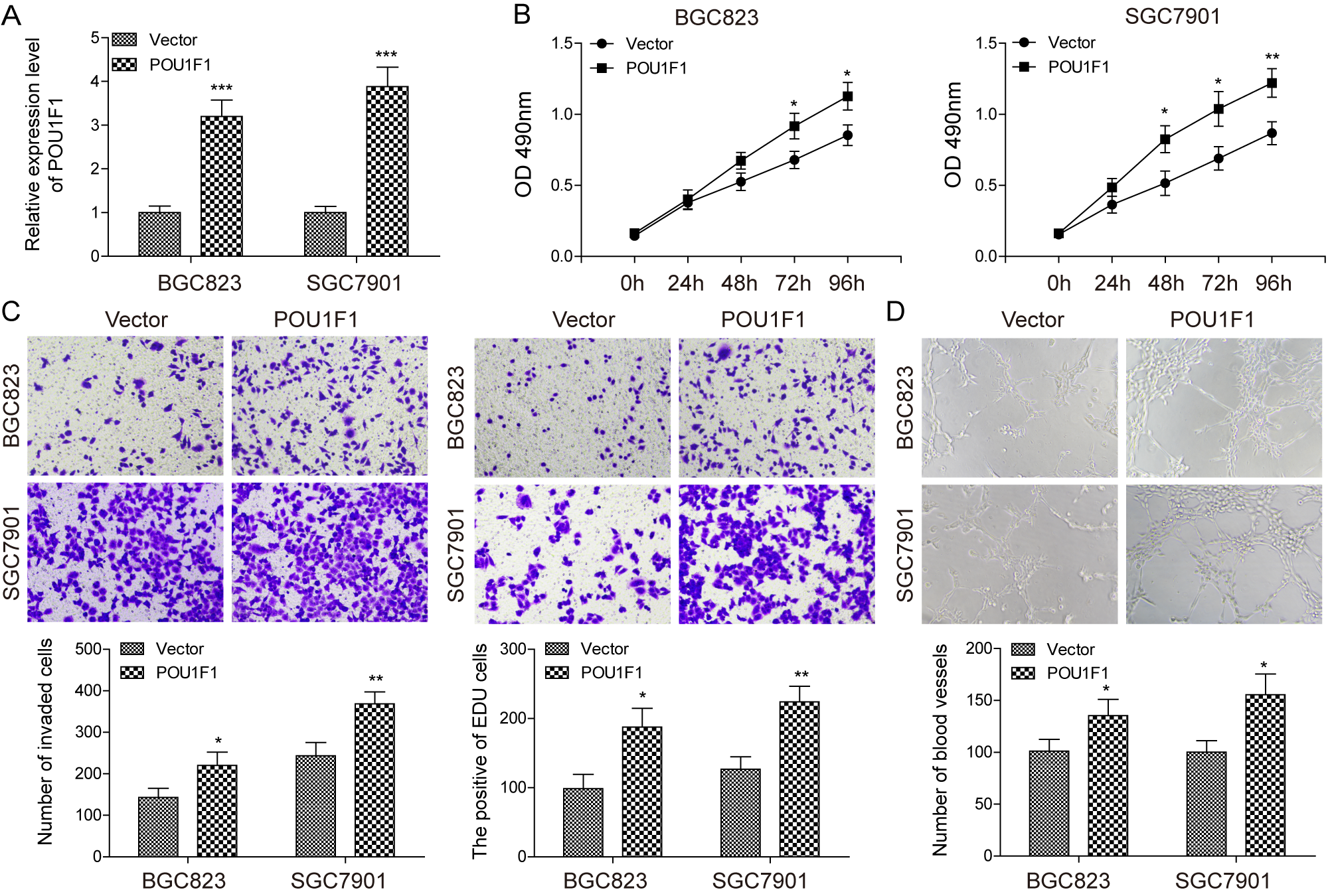

Supplement: Supplementary file 2 — FIGS1 [file 41419_2021_3703_MOESM2_ESM.tif]

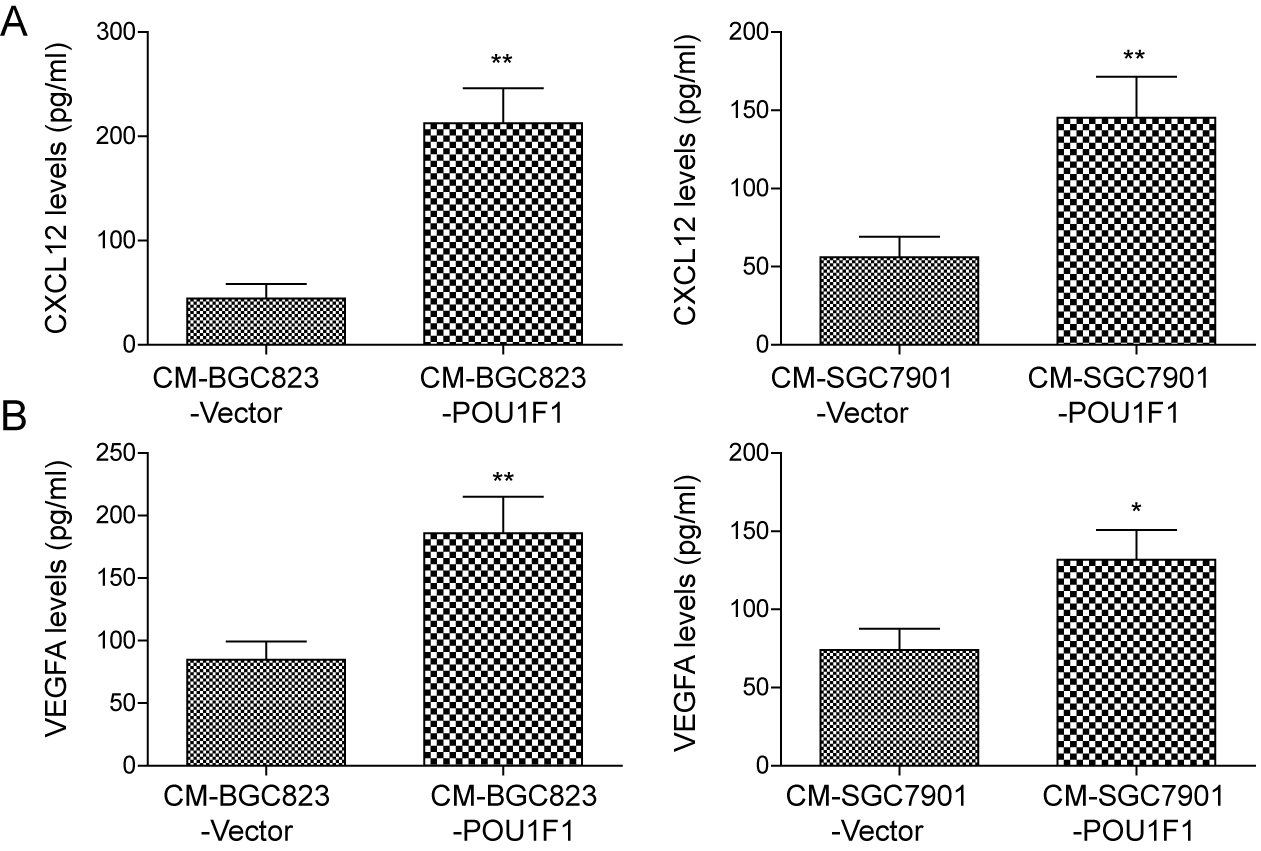

Supplement: Supplementary file 3 — FIGS2 [file 41419_2021_3703_MOESM3_ESM.tif]

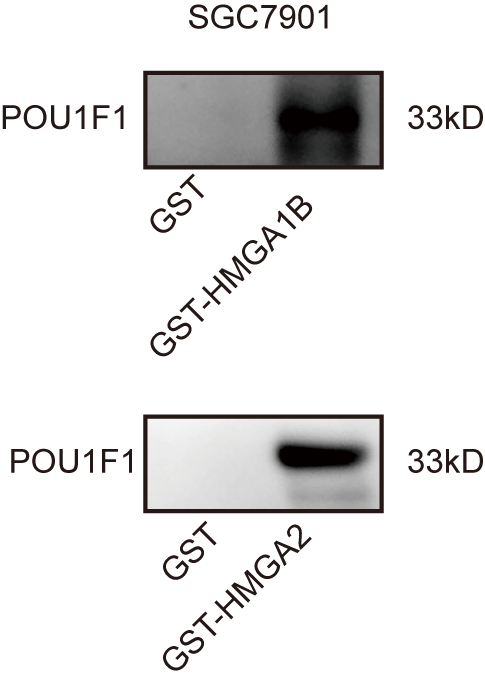

Supplement: Supplementary file 4 — FIGS3 [file 41419_2021_3703_MOESM4_ESM.tif]
